# Pyroptosis as a novel therapeutic target in glioblastoma multiforme: Mechanisms, molecular insights, and therapeutic potential

**DOI:** 10.1016/j.omtn.2026.102982

**Published:** 2026-06-11

**Authors:** Shokooh Karimi, Ahmad Reza Dehpour, Mahmoudreza Hadjighassem, Alireza Khajehnasiri, Reza Atef Yekta, Mahnaz Aghaei, Hossein Majedi, Saereh Hosseindoost

**Affiliations:** 1 Pain Research Center, Neuroscience Institute, Imam Khomeini Hospital Complex, Tehran University of Medical Sciences, Tehran, Iran; 2 Experimental Medicine Research Center, Tehran University of Medical Sciences, Tehran, Iran; 3 Department of Pharmacology, School of Medicine, Tehran University of Medical Sciences, Tehran, Iran; 4 Brain and Spinal Cord Injury Research Center, Neuroscience Institute, Imam Khomeini Hospital Complex, Tehran University of Medical Sciences, Tehran, Iran; 5 Department of Anesthesiology, Critical Care, and Pain, Dr. Ali Shariati Hospital, Tehran University of Medical Sciences, Tehran, Iran

**Keywords:** MT: Oligonucleotides: Therapies and Applications, glioblastoma multiforme, pyroptosis, inflammasome, gasdermin, caspase, noncoding RNA, targeting treatment

## Abstract

Glioblastoma multiforme (GBM) is the most malignant type of primary brain tumor. Its clinical management is challenging due to its heterogeneity, highly malignant nature, and insensitivity to standard treatments. While current strategies for GBM treatments are based on inducing apoptosis in GBM cells, some GBM tumors showed resistance to this type of cell death. Recent evidence indicates that pyroptosis is a novel, promising therapeutic method for overcoming tumor cells’ resistance to cancer treatment. This inflammatory programmed cell death type is mediated by the cleavage of gasdermin proteins. Based on the evidence, inducing pyroptosis is negatively associated with GBM growth and development; the exact molecular mechanisms and the signaling pathways underlying pyroptosis are not fully understood. This review presents the different pathways of pyroptosis and its role in GBM growth regulating and illustrates various drugs and components that modulate pyroptosis in GBM tumors. It also investigates the regulatory roles of noncoding RNAs in pyroptosis modulation in GBM tumors, providing promising therapeutic approaches that target pyroptosis as a novel strategy for GBM treatment.

## Graphical abstract

**Figure:**
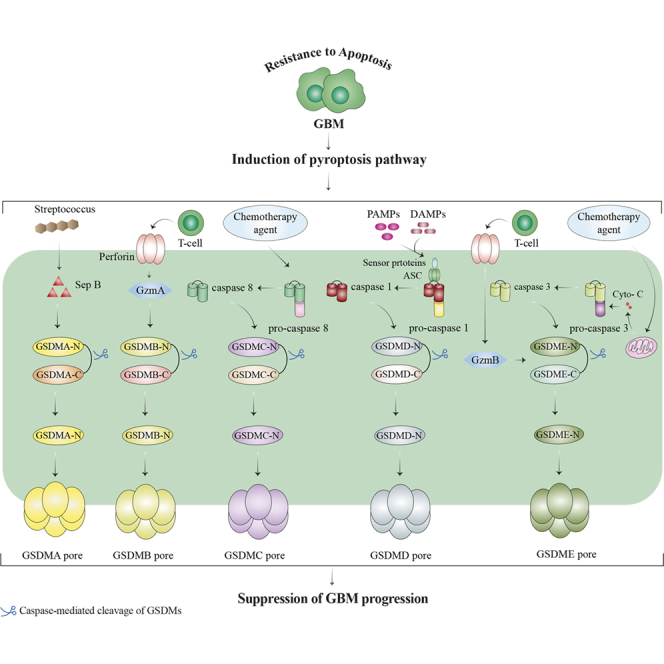

## Teaser

Pyroptosis, a novel form of programmed cell death, offers promising therapeutic potential for drug-resistant glioblastoma multiforme. This review investigates key molecular mediators and emerging strategies targeting pyroptosis, suggesting that its integration with current therapies could improve outcomes for patients with this aggressive brain tumor.

## Introduction

Glioblastoma multiforme (GBM), also known as grade IV astrocytoma, is the most prevalent and aggressive type of primary malignant brain tumor in adults, accounting for approximately 45%–50% of all primary brain tumors. Its annual incidence is 3–5 cases per 100,000 people worldwide.^1^ The main characteristics that make GBM particularly difficult to treat are its highly aggressive and invasive nature. GBM cells proliferate rapidly and infiltrate extensively into the surrounding brain parenchyma along white matter tracts, blood vessels, and perivascular spaces. This diffuse invasion prevents complete surgical resection, as tumor cells extend beyond visible tumor margins and intermingle with normal neural tissue, leading to inevitable residual disease and recurrence. Furthermore, GBM exhibits pronounced genetic and molecular heterogeneity, enabling tumor cells to adapt to therapeutic pressure and develop resistance. The infiltrative growth pattern also reduces the effectiveness of localized therapies and limits drug delivery due to the blood-brain barrier.^2^^,^^3^^,^^4^

The current standard of care for GBM includes maximal surgical resection followed by radiotherapy and concomitant and adjuvant chemotherapy with temozolomide (TMZ). Surgery aims to reduce tumor burden, although complete resection is rarely achievable due to the diffuse infiltrative nature of the disease. Radiotherapy induces DNA damage through ionizing radiation, activating intrinsic apoptotic pathways via mitochondrial outer membrane permeabilization and subsequent activation of caspase-9 and caspase-3. TMZ, an alkylating agent, promotes DNA methylation and strand breaks, leading to activation of p53-dependent apoptotic signaling cascades. In addition, emerging immunotherapeutic approaches, such as immune checkpoint inhibitors, CAR-T cell therapies, and vaccine-based strategies, aim to enhance antitumor immune responses and may induce tumor cell death through cytotoxic T-lymphocyte-mediated apoptosis.^5^

Despite these multimodal approaches, GBM remains largely incurable. Resistance mechanisms, including DNA repair, hypoxic adaptation, genetic heterogeneity, suppression of apoptotic signaling, and limited drug penetration due to the blood-brain barrier, contribute to therapeutic failure and recurrence. Resistance to apoptosis, in particular, represents a major obstacle to durable treatment responses.^6^ As a result, despite current standard treatment, many patients experience tumor recurrence, and GBM patients’ survival typically ranges from 12 to 15 months.^7^ Because most current treatments rely heavily on apoptosis induction, strategies that activate alternative programmed cell death pathways may offer therapeutic advantages.^8^^,^^9^ Pyroptosis, a distinct form of programmed cell death characterized by GSDM-mediated membrane pore formation, cellular swelling, and inflammatory cytokine release, has recently gained attention as a potential mechanism for eliminating apoptosis-resistant GBM cells.^10^ In this review, we summarize the fundamental cellular and molecular mechanisms underlying pyroptosis and discuss its regulatory pathways in GBM. We further examine key molecular mediators and emerging therapeutic strategies that may induce pyroptosis in GBM cells. Finally, we discuss the potential clinical implications of targeting pyroptosis as an alternative approach to overcome apoptosis resistance and improve therapeutic outcomes in this highly aggressive malignancy.

### Pyroptosis

Pyroptosis is a form of inflammatory programmed cell death that is distinct from other cell death pathways, such as apoptosis and necroptosis. It is characterized by the formation of pores in the cell membrane, cell swelling, and eventual lysis, leading to the release of inflammatory intracellular contents.^11^^,^^12^ In 1992, Zychlinsky et al. first described pyroptosis by observing that macrophages infected with the Gram-negative bacterium *Shigella flexneri* underwent a distinct form of cell death different from apoptosis. The term “pyroptosis” was introduced in 2001 by combining “pyro” (referring to inflammation or fever) and “ptosis” (meaning falling or programmed cell death) to describe this distinct mode of cell death. This initial definition emphasized its inflammatory nature, in contrast to the non-inflammatory process of apoptosis.^12^^,^^13^ In 2002, the inflammasome complex was first recognized for its ability to activate inflammatory caspases and process pro-IL-1β.^14^ Subsequently, Petr et al. discovered that non-canonical caspase-11 could induce cell death independently of caspase-1 during *Salmonella* infection.^15^ In the context of cancer, pyroptosis can have dual effects. On one hand, it can suppress tumor growth by inducing immunogenic cell death and stimulating anti-tumor immunity. On the other hand, pyroptosis can also promote inflammation and tumor progression under certain conditions. Therapeutically inducing pyroptosis represents a promising strategy for cancer treatment by reprogramming the tumor microenvironment and restoring the body’s antitumor immune response.^16^

Pyroptosis is triggered by various pathological stimuli such as infection, stroke, myocardial infarction, and cancer. It is mediated by the gasdermin (GSDM) family of proteins, which consists of six members (gasdermins A–E and DFNB59). GSDMs belong to the pore-forming protein family and have two domains: an N-terminal domain and a C-terminal domain connected by a flexible linker peptide. Upon activation, the cleaved N-terminal domain is responsible for inducing pyroptosis.^17^^,^^18^ GSDMs are cleaved by inflammatory caspases or granzymes, resulting in the release of the N-terminal domain. The N-terminal fragment targets the cell membrane and induces pore formation by binding to phosphatidylinositol, phosphatidic acid, and phosphatidylserine. Pore formation leads to osmotic swelling, membrane rupture, and the release of inflammatory cytokines. Extensive membrane perforation compromises membrane integrity, resulting in lytic cell death and the release of cytoplasmic contents that further amplify inflammatory signaling. Ultimately, multiple pro-inflammatory cytokines are released into the extracellular space, triggering a robust inflammatory response.^19^^,^^20^

For a long time, pyroptosis was thought to be a form of monocyte cell death induced by caspase-1.^21^ However, recent studies have expanded our understanding of pyroptosis. It has been reported that caspase-1 or caspase-11/4/5 is activated during this process, leading to the cleavage of gasdermin D (GSDMD) and the formation of pores in the cell membrane.^22^ Gasdermin E (GSDME) is another crucial mediator in the pyroptosis pathway. Certain chemotherapeutic agents can induce pyroptosis by activating caspase-3, which cleaves GSDME. Caspase-8 is also induced through GSDME activation, affecting inflammasome function.^23^^,^^24^ Moreover, granzyme B can directly cleave GSDME and activate pyroptosis, further stimulating the antitumor immune response and inhibiting tumor growth.^25^ Additionally, granzyme A in cytotoxic lymphocytes can enter target cells via perforin and induce pyroptosis by cleaving GSDMB at specific sites, further refining our understanding of this process.^26^ Recent studies have demonstrated that pyroptosis is an essential pathway in cancer progression and antitumor immunity. However, there is still limited knowledge regarding its role in GBM.^10^

### Molecular mechanisms of pyroptosis

**Figure 1:**
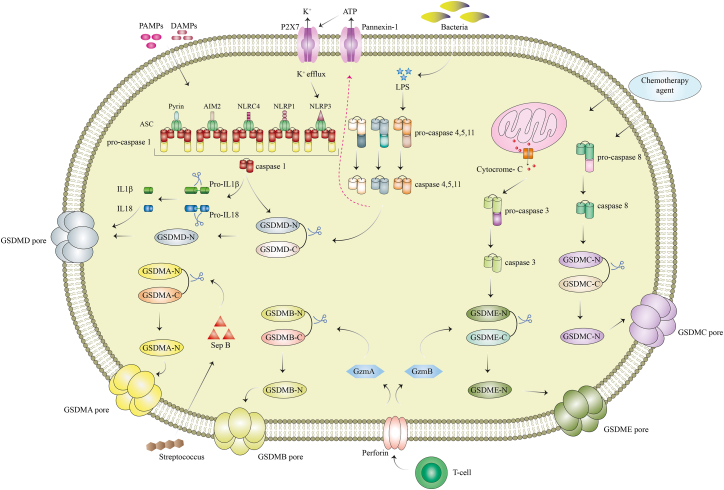
A schematic of main pyroptosis pathway

Pyroptosis is activated through several distinct pathways, primarily categorized into the canonical inflammasome pathway, the non-canonical inflammasome pathway, a newly identified GSDME-mediated pathway, and a granzyme-mediated pathway. The canonical pathway relies on the activation of caspase-1 through inflammasome complexes formed by pattern recognition receptors, which leads to the cleavage of GSDMD and the release of pro-inflammatory cytokines. In contrast, the non-canonical pathway involves the direct activation of caspases 4, 5, or 11 by intracellular lipopolysaccharide (LPS), resulting in GSDMD cleavage. The GSDME-mediated pathway is mostly characterized by the activation of caspase-3, which cleaves GSDME and triggers pyroptosis in specific cell types. Finally, the granzyme-mediated pathway involves serine proteases released by cytotoxic lymphocytes, which can induce pyroptosis in target cells. Collectively, these pathways show the diverse mechanisms through which pyroptosis can be initiated and its significant role in inflammation and immune responses (Figure 1).^27^

### Canonical inflammasome pyroptosis pathway

The canonical inflammasome pathway is a critical mechanism in the innate immune response, characterized by the activation of caspase-1 through various inflammasomes. Inflammasomes comprise three core components: a sensor protein, an adaptor protein, and the effector protein caspase-1. Upon recognizing pathogen-associated molecular patterns (PAMPs) or damage-associated molecular patterns (DAMPs), sensor proteins, such as the NOD-like receptor (NLR) family, absent in melanoma 2 (AIM2), or pyrin, recruit the apoptosis-associated speck-like protein containing a CARD (ASC) adaptor protein and caspase-1, leading to caspase-1 activation. Activated caspase-1 cleaves pro-IL-1β and pro-IL-18 into their mature, active forms and induces pyroptosis by cleaving GSDMD. The N-terminal fragment of GSDMD forms pores in the plasma membrane, leading to pyroptotic cell death. These pores also facilitate the release of mature interleukin (IL)-1β and IL-18, thereby amplifying the inflammatory response.^28^

The inflammasome-mediated cleavage of GSDMD and subsequent cell lysis represent key regulatory pathways of pyroptosis. Pattern recognition receptors (PRRs), primarily from the NLR family, AIM2, pyrin, and CARD8, recognize PAMPs and DAMPs. Upon activation by these signals, the PRRs oligomerize and recruit ASC, forming canonical inflammasome complexes. This process involves the autoproteolytic activation of pro-caspase-1 into active caspase-1, which triggers a cascade of inflammatory responses. During inflammasome activation, ASC can form insoluble polymers known as ASC specks, which serve as platforms that amplify caspase-1 activation.^18^ While ASC is essential for assembly of several inflammasomes, certain PRRs like NLRP1b and NLRC4 can directly interact with pro-caspase-1, allowing for caspase-1 activation even in the absence of ASC. However, the efficiency of cleaving inflammatory cytokines such as IL-1β and IL-18 is significantly enhanced in the presence of ASC. The activation of caspase-1, whether through ASC-dependent or -independent mechanisms, leads to GSDMD cleavage and pore formation in the plasma membrane. Pore formation allows the release of mature IL-1β and IL-18 into the extracellular space and causes pyroptosis due to the influx of ions and water, leading to cell swelling and lysis.^29^

### Non-canonical inflammasome pyroptosis pathway

The non-canonical pyroptosis pathway is primarily activated by caspase-4 and caspase-5 in humans and caspase-11 in mice in response to intracellular LPS. Unlike the canonical pathway, this pathway does not rely on upstream sensory complexes. Instead, these caspases can be activated directly through binding to intracellular LPS via their N-terminal CARD domain, and activated caspase-4/5/11 cleaves GSDMD into N-GSDMD, which oligomerizes and translocates to the plasma membrane, forming pores that lead to cell lysis. While caspase-4/5/11 do not directly cleave pro-IL-1β or pro-IL-18 directly, they can facilitate the maturation and secretion of these cytokines through the NLRP3/caspase-1 pathway in specific cell types.^30^ Moreover, oxidized phospholipids, such as oxidized 1-palmitoyl-2-arachidonoyl-sn-glycero-3-phosphorylcholine (oxPAPC), act as TLR4 agonists and inhibit LPS binding to caspase-4/11. This competition can reduce non-canonical inflammasome activation in macrophages, although dendritic cells appear to be unaffected by this interaction.^31^

The cleavage of GSDMD by caspase-4/5/11 results in potassium (K+) efflux, which is critical for the assembly of the NLRP3 inflammasome, ultimately leading to pyroptosis.^32^ Pannexin-1 has been identified as a key protein in mediating pyroptotic cell death in the non-canonical pathway. Under LPS stimulation, activated caspase-11 can cleave and activate Pannexin-1, promoting ATP release and subsequent activation of the P2X7 signaling, which further promotes pyroptosis. Interestingly, studies have shown that murine bone-marrow-derived macrophages (BMDMs) lacking Pannexin-1 can still induce K+ efflux and activate the NLRP3 inflammasome, resulting in caspase-1 cleavage independently of P2X7. This suggests that selective K+ channels play a significant role in modulating non-canonical NLRP3 activation. Moreover, knockout of Pannexin-1 in mice has been linked to protection against endotoxin shock, showing its regulatory function in the inflammatory response.^33^

### GSDME-mediated pyroptosis pathway

GSDME, also known as DFNA5, is a member of the GSDM protein family that can induce pyroptosis. The GSDME-mediated pyroptosis pathway is distinct from the GSDMD-mediated pathway. In contrast to GSDMD-dependent pyroptosis, which involves inflammasome assembly, GSDME-mediated pyroptosis occurs independently of inflammasome formation. In this context, caspase-3 plays a central role in activating GSDME and initiating pyroptosis through a mechanism distinct from classical inflammasome pathways. During apoptotic stimuli, such as chemotherapy-induced stress, caspase-3 cleaves GSDME at its linker region, generating an N-terminal fragment (GSDME-N) and a C-terminal fragment (GSDME-C). GSDME-N targets the plasma membrane, inducing pore formation, which leads to cell swelling, lysis, and the release of inflammatory contents, ultimately inducing pyroptosis. The release of pro-inflammatory cytokines, such as IL-1β and IL-18, amplifies the inflammatory response, contributing to the antitumor immune response and the progression of inflammatory diseases. GSDME-mediated pyroptosis promotes antitumor immunity by converting caspase-3-dependent apoptosis into pyroptosis, releasing pro-inflammatory cytokines and transforming the tumor microenvironment from a “cold” to a “hot” state. However, GSDME is expressed in nearly all body tissues and immune cells, which can exacerbate chemotherapy toxicity and partially block the immune response. GSDME expression is transcriptionally regulated by the signal transducer and activator of transcription 3 (STAT3), which directly binds to its promoter region and modulates GSDME expression. This regulation is crucial for the induction of pyroptosis in various inflammatory conditions.^34^^,^^35^^,^^36^^,^^37^

### Pyroptosis mediated by other gasdermins

GSDMA has a single transcriptional copy in the human genome, while mice have three (Gsdma1, Gsdma2, and Gsdma3) due to gene duplications during evolution.^38^ The N-terminal fragments of GSDMA (GSDMA-NT) and Gsdma3 (Gsdma3-NT) showed similar pore-forming activity to the GSDMD-NT and GSDME-NT. These N-terminal fragments can bind to cell membrane lipids, such as cardiolipin or phosphoinositide, and then induce pore formation, leading to pyroptosis.^39^ While the specific cleavage mechanisms of GSDMA in humans are incompletely characterized, studies have demonstrated that GSDMA can be cleaved by particular proteases. For example, in *Streptococcus pyogenes* infections, the cysteine protease streptococcal pyrogenic exotoxin B (SpeB) triggers the release of active N-terminal fragments of GSDMA by cleaving it after Gln246, initiating pyroptosis and playing a crucial role in immune defense against invasive skin infections.^40^

Gasdermin C (GSDMC) has been confirmed to play a significant role in pyroptosis. It was initially identified as highly expressed in metastatic melanoma cells, referred to as MLZE (melanoma-derived leucine zipper-containing extranuclear factor). Recent studies have further clarified the function of GSDMC.^41^ For example, research by Hou et al. demonstrated that GSDMC can be cleaved by caspase-8 under hypoxic conditions. This cleavage event can switch tumor necrosis factor alpha (TNF-α)-induced apoptosis to pyroptosis in MDA-MB-231 breast cancer cells in the context of death receptor signaling.^42^ Furthermore, α-ketoglutarate (α-KG) has been shown to effectively penetrate cell membranes and induce tumor pyroptosis, suggesting potential antitumor activity. In an acidic environment, α-KG is converted by the metabolic enzyme malate dehydrogenase 1 (MDH1) into L-2-hydroxyglutarate (L-2HG). This conversion increases reactive oxygen species (ROS) levels, which induce death receptor-6 (DR6). DR6 promotes membrane oxidation, polymerization, and endocytosis, forming receptosomes. Within the receptosomes, endocytic DR6 recruits pro-caspase-8 via the mediation of the adaptor protein Fas-associated protein with death domain (FADD). GSDMC is also recruited to the receptosomes, where it undergoes cleavage by active caspase-8. This cleavage results in the release of the N-terminal fragment of GSDMC, which then inserts into the cell membrane, forming pores and ultimately triggering pyroptosis.^43^

Moreover, chemotherapeutic agents activate the caspase-8/GSDMC-mediated pyroptosis pathway in breast cancer cells.^12^ These findings show the complex interplay between different signaling pathways and metabolic processes in regulating cell death mechanisms, particularly in cancer. Understanding the role of GSDMC in switching between apoptosis and pyroptosis may provide insights into potential therapeutic strategies targeting tumor cell death.

Gasdermin B (GSDMB) is the least studied member of the GSDM family. However, emerging evidence suggests that it plays an important role in restoring epithelial barrier function and resolving inflammation. Interferon gamma (IFN-γ) has been shown to enhance the expression of GSDMB and promote pyroptosis.^44^^,^^45^ GSDMB is located at the same chromosomal site (17q21.1) as GSDMA in humans, but the GSDMB gene is absent in mice. It is specifically expressed in the epithelial tissues of the esophagus and gastrointestinal tract and in the bronchial epithelium of the lungs affected by asthma.^46^ Additionally, GSDMB is found in liver, neuroendocrine, and immune cells.^47^^,^^48^^,^^49^ The GSDMB-NT domain can trigger pyroptosis in HEK293T cells, and research by Panganiban et al. revealed that caspase-1 cleaves full-length GSDMB at position Asp236.^50^ In contrast, a study by Chao et al. suggests that GSDMB is not a substrate for the inflammatory caspases (caspase-1, -4, -5, or -11), as it lacks the specific interdomain junction regions required for cleavage.^51^ Overall, the role of GSDMB in pyroptosis remains controversial, and further research is needed to clarify the mechanisms by which caspases interact with GSDMB.

### Granzyme-mediated pyroptosis pathway

This pathway is characterized by the action of granzymes A and B, which are serine proteases released by cytotoxic T lymphocytes and natural killer (NK) cells.^52^ Granzyme A cleaves GSDMB, while granzyme B cleaves GSDME, resulting in pyroptosis. This pathway is notable for its caspase-independent mechanism of action and its role in enhancing antitumor immunity. Before recent discoveries, it was believed that the cytotoxic capabilities of cytotoxic T lymphocytes and NK cells depended solely on the granzyme-induced apoptosis of target cells. However, further research has revealed that pyroptosis is an additional mechanism these cytotoxic lymphocytes employ to eliminate GSDMB-expressing target cells.^26^ Granzyme A cleaves GSDMB at specific sites, causing pore formation, cellular swelling, and cell rupture, leading to pyroptotic cell death. Interestingly, IFN-γ has been shown to upregulate the expression of GSDMB and enhance this pyroptotic response, potentially contributing to improved antitumor immunity and the clearance of virus-infected cells.^53^

Liu et al. found that CAR T cells rapidly activate caspase-3 in B cell leukemia cells and other target cells through the release of granzyme B. This activation subsequently triggers the caspase-3/GSDME-mediated pyroptotic pathway, leading to extensive pyroptosis. Consequently, the inflammatory factors released from pyroptosis further activate the caspase-1/GSDMD pathway and the mitogen-activated protein kinase (MAPK)-nuclear factor κB (NF-κB) signaling pathway, resulting in cytokine release syndrome. The studies collectively confirm the critical role of the granzyme A/GSDMB and granzyme B/GSDME pathways in anti-tumor immune processes.^54^

The expression of GSDMB, unlike other GSDMs, in HEK293T cells induces pyroptotic cell death mediated by NK cells. This process was accompanied by the cleavage of GSDMB within its interdomain region. However, inhibiting the perforin-granzyme pathway blocked these pyroptotic processes in HEK 293T cells. *In vitro*, analysis of all five human granzymes revealed that granzyme A effectively cleaves GSDMB, primarily at the Lys244 site within the interdomain linker. This cleavage exposed the pore-forming capability of GSDMB. When granzyme A was introduced into cells reconstituted with GSDMB via electroporation or perforin, it triggered significant pyroptosis along with interdomain cleavage of GSDMB. These effects were reduced by a K229A/K244A (KK/A) mutation of GSDMB, where lysine (K) was substituted with alanine (A) at positions 229 and 244, respectively. In cells that typically undergo apoptosis when exposed to granzyme A, the introduction of GSDMB, which can be cleaved by granzyme A, transformed the mode of cell death from apoptosis to pyroptosis. The pyroptotic killing by NK cells was inhibited by the KK/AA mutation in GSDMB and a knockdown of granzyme A expression.^26^

Moreover, another study showed that granzyme proteases contribute to NK-cell-induced pyroptosis through the cleavage of GSDME. Granzyme B, released by NK cells and CD8^+^ T cells, directly cleaves GSDME at aspartate 270 (D270) to induce pyroptosis in tumor cells. This cleavage activates GSDME’s pore-forming ability, triggering inflammatory cell death. The resulting pyroptosis releases pro-inflammatory signals that recruit and activate immune cells, including tumor-associated macrophages, which enhance phagocytosis of tumor debris. This process creates a self-reinforcing anti-tumor immune response: pyroptotic cells attract additional cytotoxic lymphocytes to the tumor microenvironment, amplifying the destruction of cancer cells. The study indicated that GSDME-dependent tumor suppression relies primarily on this immune-mediated mechanism, as shown by the loss of antitumor effects in models lacking perforin or lymphocytes. By bridging cytotoxic cell function and inflammatory cell death, the granzyme B-GSDME axis emerges as a critical pathway for activating systemic antitumor immunity and inhibiting cancer progression.^55^

### Similarities and differences between apoptosis and pyroptosis

Apoptosis and pyroptosis exhibit distinct morphological and molecular characteristics. However, both processes are mediated by caspases. Certain caspases, such as caspase-3, are involved in both processes, indicating shared molecular mechanisms. While apoptosis can release pro-inflammatory signals under certain conditions, pyroptosis is primarily inflammatory, actively releasing cytokines such as IL-1β and IL-18. Apoptosis is characterized by cell shrinkage, chromatin condensation, and the formation of apoptotic bodies, leading to a non-inflammatory outcome.^56^ In contrast, pyroptosis involves cell swelling, membrane rupture, and the release of intracellular contents, provoking inflammation. The outcomes of these processes show their fundamental differences. Apoptosis safely removes cells without eliciting inflammation, which is crucial for tissue homeostasis. In contrast, pyroptosis is a lytic form of cell death that promotes inflammation and can damage tissue if dysregulated. Pyroptosis shares features with apoptosis, such as nuclear condensation and DNA damage, but exhibits unique traits, including a distinct type of DNA damage. Pyroptotic cells maintain intact nuclei despite chromatin condensation and DNA fragmentation, showing lower intensity in DNA damage than apoptotic cells. In apoptosis, DNA damage relies on caspase-activated DNase (CAD) and its inhibitor (ICAD), but CAD is unnecessary in pyroptosis, even though caspase-1 can cleave CAD. Additionally, pyroptosis is marked by inflammation-associated pore formation, leading to cell swelling and osmotic lysis.^57^^,^^58^ While both processes involve caspase activation, the specific caspases differ. Apoptosis activates caspases 2, 3, 6, 7, 8, 9, 10, and 12, while pyroptosis is induced by inflammatory caspases, including caspase 1, 3, 4, 5, 8, 9, and murine caspase-11.^59^^,^^60^^,^^61^^,^^62^ Caspase-3, usually associated with apoptosis, can also participate in pyroptosis. Lastly, apoptotic cells are sensitive to ATP levels, with DNA damage activating poly(ADP-ribose) polymerase (PARP) and leading to ATP depletion. During apoptosis, caspases cleave PARP to limit excessive ATP consumption, while PARP is not involved in pyroptosis.^63^

Although apoptosis and pyroptosis are traditionally considered distinct forms of programmed cell death, increasing evidence indicates mechanistic convergence, particularly at the level of executioner caspases. This overlap provides a rationale for targeting shared molecular nodes to simultaneously engage both pathways and overcome apoptosis resistance in GBM.^64^^,^^65^^,^^66^ A central convergence point is caspase-3, which in GSDME-expressing cells cleaves GSDME to generate a pore-forming N-terminal fragment, thereby converting apoptotic signaling into pyroptosis. This positions GSDME as a molecular switch of cell fate and a promising therapeutic target for dual-pathway activation.^39^^,^^57^^,^^67^^,^^68^^,^^69^^,^^70^

Preclinical studies further support this concept. For example, benzimidazole compounds induce both mitochondria-dependent apoptosis and NF-κB/NLRP3/GSDMD-mediated pyroptosis in GBM, leading to significant tumor suppression *in vivo*.^64^ In addition, shared upstream stress signals such as oxidative stress and mitochondrial dysfunction can activate both apoptotic and pyroptotic pathways, suggesting that modulation of cellular stress responses may engage multiple cell death programs simultaneously.^71^^,^^72^^,^^73^ Moreover, oncogenic signaling pathways, including NF-κB, STAT3, and PI3K/AKT/mTOR, suppress both apoptotic and pyroptotic machinery in GBM. Their inhibition can restore pro-death signaling and sensitize tumor cells to multiple forms of programmed cell death.^64^^,^^65^^,^^74^^,^^75^ Epigenetic regulation and ncRNAs also coordinately suppress apoptotic genes and gasdermins, and targeting these regulators may reactivate both pathways in parallel.^76^^,^^77^^,^^78^

Collectively, these shared mechanisms support a shift from targeting individual cell death pathways toward network-level modulation of cell death susceptibility. Simultaneous activation of apoptosis and pyroptosis may reduce therapeutic resistance and improve treatment efficacy in GBM. Identifying shared regulatory nodes therefore represents a promising strategy for dual-pathway therapeutic development.

### Apoptotic caspase-mediated pyroptosis pathway

Caspase-dependent signaling represents a key mechanistic link between apoptosis and pyroptosis. In addition to inflammatory caspases such as caspase-1, -4, -5, and murine caspase-11, several apoptotic caspases can trigger pyroptosis under specific conditions. Chemotherapeutic agents typically induce caspase-3-dependent apoptosis; however, in cells expressing gasdermins, this signaling can be redirected toward pyroptosis.

A major example is GSDME, where activated caspase-3 cleaves GSDME to generate its pore-forming N-terminal fragment, thereby converting apoptotic cell death into pyroptosis.^39^^,^^57^^,^^67^^,^^68^^,^^69^^,^^70^ This mechanism has been observed with conventional chemotherapeutic agents such as cisplatin, which induce pyroptosis through caspase-3-mediated GSDME cleavage.^79^^,^^80^^,^^81^

Similarly, mitochondrial stress pathways, including Bax activation, cytochrome *c* release, and caspase-9/caspase-3 activation, can also converge on GSDME cleavage, linking oxidative-stress-induced apoptosis to pyroptotic cell death.^82^

Moreover, caspase-8 serves as another important bridge between these pathways. It can cleave GSDMD during inflammatory signaling, such as Yersinia infection.^83^^,^^84^ When transforming growth factor-β-activated kinase 1 (TAK1) is inhibited by the Yersinia effector YopJ, the lysosomal Rag-Ragulator complex serves as a platform for activating an FADD/receptor-interacting serine-threonine protein kinase 1 (RIPK1)/caspase-8 complex, ultimately triggering pyroptosis. Furthermore, caspase-8 can also cleave GSDMC, releasing the N-terminus of GSDMC to form pores in the cancer cell membrane.^85^

**Figure 2:**
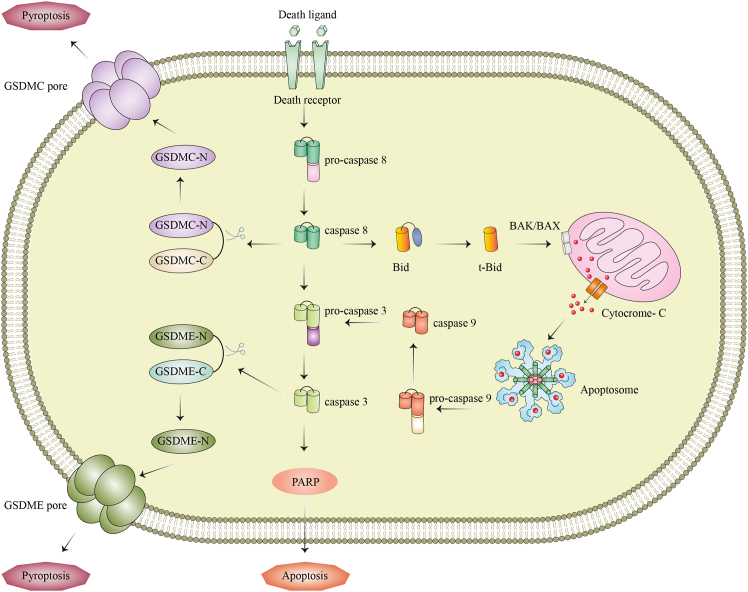
A schematic of apoptotic caspase-mediated pyroptosis pathway

In addition, caspase-3, -6, and -7 have been shown to cleave GSDMB, removing its C-terminal inhibitory domain and releasing its pore-forming N-terminal fragment, thereby inducing pyroptosis.^86^ Collectively, these findings demonstrate that both apoptotic and inflammatory caspases can act on different gasdermin family members, enabling multiple routes for converting apoptotic signaling into pyroptosis (Figure 2).^86^

### Pyroptosis in glioblastoma multiforme

Pyroptosis has emerged as an important regulator of GBM pathogenesis and progression. Although its role has been extensively studied in other tumor types, its contribution to GBM biology is only beginning to be elucidated.^87^^,^^88^ Recent studies have identified a set of pyroptosis-associated genes (PAGs) that are differentially expressed in GBM compared with normal brain tissue, with several genes significantly upregulated in tumor samples. For instance, one study reported 29 differentially expressed PAGs in GBM, of which 18 were upregulated.^87^ The dual role of pyroptosis in GBM is increasingly recognized. On the one hand, induction of pyroptosis can directly promote tumor cell death, representing a potential strategy to overcome resistance to apoptosis-based therapies. On the other hand, pyroptosis-associated cell lysis and the release of intracellular contents may exert complex effects on tumor progression. Nevertheless, the ability of pyroptosis to bypass apoptosis resistance and modulate the tumor microenvironment shows its potential as a therapeutic target in GBM.^36^^,^^87^^,^^89^^,^^90^^,^^91^

Pyroptosis has emerged as a highly relevant alternative form of programmed cell death in GBM, particularly in the context of apoptosis resistance, a hallmark of this malignancy. Resistance to apoptosis arises from multiple molecular alterations, including frequent p53 mutations, dysregulation of BCL-2 family proteins, hyperactivation of PI3K/AKT signaling, and epigenetic silencing of pro-apoptotic genes. These changes impair caspase-dependent apoptotic execution and reduce the effectiveness of therapies such as chemotherapy and radiotherapy, which primarily rely on apoptosis induction.^92^^,^^93^ Clinically, despite aggressive multimodal treatment, nearly all GBM tumors recur, reflecting widespread resistance in the majority of patients.^7^ Both intrinsic and acquired resistance mechanisms contribute to therapeutic failure in the vast majority of patients. Therefore, there is a need for alternative mechanisms that operate independently of the classical apoptotic pathway.^94^^,^^95^ In contrast to apoptosis, pyroptosis provides a mechanistically distinct route for tumor cell elimination. Notably, in GSDME-expressing GBM cells, activation of caspase-3 can redirect apoptotic signaling toward pyroptosis. This functional switch enables pyroptosis to act as a compensatory cell death mechanism when apoptosis is suppressed or incomplete.^35^^,^^36^

Beyond serving as an alternative pathway, pyroptosis may also enhance the efficacy of current GBM therapies. Several agents, including TMZ, arsenic trioxide, and AT7519, can induce pyroptosis either directly or indirectly through ROS accumulation, caspase activation, and GSDM cleavage.^65^^,^^96^ In apoptosis-resistant GBM cells, redirecting therapy-induced stress toward pyroptosis can increase tumor cell killing.^97^ Moreover, the inflammatory nature of pyroptosis may remodel the tumor microenvironment by releasing DAMPs and pro-inflammatory cytokines, thereby enhancing immune cell recruitment and activation. This suggests that pyroptosis induction could synergize with radiotherapy and emerging immunotherapeutic strategies by converting the typically immunosuppressive GBM microenvironment into a more immunologically active state.^98^ Collectively, these findings support pyroptosis as both an alternative cell death mechanism in apoptosis-resistant GBM and a potential therapeutic sensitizer. Further investigation into the molecular determinants of pyroptosis sensitivity will be essential for translating these insights into clinical applications.

### GBM-associated signaling pathways involved in pyroptosis regulation

GBM is driven by complex and highly interconnected signaling networks that regulate tumor growth, survival, immune evasion, and therapeutic resistance. Recent evidence indicates that many of these pathways converge on pyroptosis, positioning it as a critical node in GBM biology and a promising therapeutic target. One of the most extensively studied axes is the NF-κB/NLRP3 inflammasome pathway. NF-κB signaling is frequently dysregulated in GBM and promotes tumor cell survival and invasion by driving the transcription of pro-inflammatory mediators, including pro-IL-1β, pro-IL-18, NLRP3, and caspase-1. Consequently, aberrant NF-κB activation may disrupt the priming phase of pyroptosis.^26^^,^^99^ The STAT3 pathway is another critical regulator of tumor progression. STAT3 is constitutively dysregulated in GBM and contributes to proliferation, immune suppression, and treatment resistance. Emerging evidence suggests that STAT3 negatively regulates pyroptosis by suppressing inflammasome activation and gasdermin expression. Pharmacological inhibition of STAT3, including indirect targeting via epigenetic regulators such as EZH2, enhances NLRP3 and GSDMD expression and promotes pyroptotic cell death. Agents such as DZNep and Xihuang Pill exert anti-GBM effects by targeting the EZH2-STAT3 axis.^100^^,^^101^

The PI3K/AKT/mTOR pathway, one of the most frequently dysregulated pathways in GBM, also intersects with pyroptosis. This pathway supports cell survival, metabolic adaptation, and resistance to oxidative stress. Its inhibition promotes ROS accumulation, which can activate inflammasomes and caspase-dependent cell death. For example, melatonin, particularly in combination with TMZ, suppresses the insulin-like growth factor 1 (IGF-1)/AKT/mTOR pathway and downstream Nrf2-mediated antioxidant responses, thereby enhancing ROS-mediated pyroptosis.^102^^,^^103^^,^^104^ Elevated ROS levels represent a central mechanism linking GBM metabolism to pyroptosis. ROS can activate both inflammasome-dependent (NLRP3/caspase-1/GSDMD) and inflammasome-independent (caspase-3/GSDME) pathways. Several compounds, including TMZ, kaempferol, aloe-emodin, arsenic trioxide, and lidocaine, induce oxidative stress that promotes caspase activation and GSDMD cleavage, ultimately triggering pyroptotic cell death.^105^^,^^106^

The caspase-3/GSDME axis is particularly relevant in GBM, as it enables the redirection of apoptosis toward pyroptosis. In GSDME-expressing cells, apoptosis-inducing agents can trigger caspase-3-mediated GSDME cleavage, resulting in pyroptotic cell death. Compounds such as AT7519, arsenic trioxide, galangin, aloe-emodin, and lidocaine induce pyroptosis via this mechanism in GBM cells.^96^^,^^107^^,^^108^^,^^109^^,^^110^ Epigenetic regulation represents an additional layer of control. Epigenetic modifiers, including EZH2, suppress the transcription of pyroptosis-related genes, thereby contributing to immune evasion and tumor persistence. Targeting these regulators can restore pyroptotic signaling and sensitize tumor cells to inflammatory cell death.^74^

Collectively, these findings demonstrate that pyroptosis in GBM is tightly regulated by multiple oncogenic and inflammatory signaling pathways. Accordingly, Table 1 summarizes key therapeutic agents, their primary molecular targets, and the associated signaling pathways involved in pyroptosis regulation, providing a comprehensive framework for understanding how pyroptosis can be therapeutically exploited in GBM.

**Table 1:** Agents regulating pyroptosis in glioblastoma multiforme

Reference	Role in pyroptosis	Result	Human/Animal	Model	Drug
Liu, J. et al.^111^	promotion	upregulating GSDMD, IL-1β, and cleaved-caspase-1 expression	U87 and U251 cells	*in vitro*	TMZ
Yao, J. et al.^112^	promotion	upregulating NLRP3, caspas-1, and N-GSDMD, downregulating caspase-3	U87 and U251 cells	*in vitro*	TMZ
Ren, L.-w. et al.^64^	promotion	increasing Myd88, NF-κB, IL-1β, NLRP3, caspase-1, and GSDMD-N expression. Inhibiting GBM cell proliferation. reducing tumor growth	U87 and U251 cells/BALB/c nude mice	*in vitro*/*in vivo*	benzimidazole compounds (flubendazole, mebendazole, fenbendazole)
Chen, S. et al.^113^	promotion	increasing ROS generation, GSDME cleavage, IL-1β, and ASC expression. Reducing mitochondrial membrane potential and GBM proliferation. suppressing tumor size	U87 and U251 cells/BALB/c nude mice	*in vitro*/*in vivo*	kaempferol
Pan, H. et al.^114^	promotion	reducing cell survival rate; enhancing ROS, NLRP2, cleaved caspase-1, IL-1β, and IL-18 levels; reducing Nrf2 expression inhibiting Nrf2 expression and tumor proliferation	U87, U251, A172, U118, T98, and human primary GBM/BALB/c nude mice	*in vitro*/*in vivo*	melatonin
Li, W. et al.^115^	promotion	inhibition of cell proliferation, enhancement of IL-1β, IL-18, and NLRP3 expressions. Reduction of tumor volume and the tumor mass	U87, U251 and T98G cells/BALB/c nude mice	*in vitro*/*in vivo*	dimethoxycanthin-6-one
Zhao, W. et al.^96^	promotion	inhibiting cell viability and proliferation. Elevating GSDME-N levels inhibiting tumor growth	U87 and U251 cells/BALB/c nude mice	*in vitro*/*in vivo*	AT7519
Yu, D. et al.^74^	promotion	inhibiting cell viability. Increasing NLRP3 and GSDMD levels. Reducing p-STAT3 levels. Increasing IL-1β and IL-18 expression	U87, LN229, A172, and H4 cells	*in vitro*	DZNep
Tang, N. et al.^116^	promotion	downregulating POU4F1 and STAT3 levels. Reducing cell viability. Increasing LDH, IL-1β and IL-18, cleaved caspase-1, ASC, and NLRP3 levels suppressing tumor growth	U251 and SHG-44 cells/BALB/c nude mice	*in vitro*/*in vivo*	XHP
Wang, J. et al.^107^	promotion	increasing cleaved GSDME, cleaved caspase-3, and LDH release	U251 cells	*in vitro*	ATO
Kong, Y. et al.^108^	promotion	inhibiting the viability and proliferation of cells. Increasing cleaved GSDME and LDH release	U87, U25, and A172 cells	*in vitro*	galangin
Wu, Y. et al.^117^	promotion	suppressing the proliferation, migration, and invasion of cells. Inhibiting NLRP3 levels inhibiting tumor growth	U87 and U25 cells/BALB/c nude mice	*in vitro*/*in vivo*	IBC
Fang, X. et al.^109^	promotion	inhibiting cell proliferation. Increasing cleaved caspase-3, cleaved GSDME, and LDH levels	U87, U25, GL261, and DBTRG cells/GL261-Luc tumor-bearing C57BL/6 J mice	*in vitro*/*in vivo*	AE
Zhou, B. et al.^110^	promotion	inhibiting cell proliferation. Upregulating cleaved caspase-3 and cleaved GSDME levels. Activating CaMKII, increasing *p*-TRPV1 and calcium overload	U87 cells	*in vitro*	lidocaine
Yang, S. et al.^118^	inhibition	downregulating caspase-1, NLRP3, and IL-1β levels. Inhibiting cell proliferation and invasion	U87 and U25 cells	*in vitro*	simvastatin

### Components that regulate pyroptosis in glioblastoma multiforme

Several agents have been identified that can induce pyroptosis in GBM. These pyroptosis-inducing compounds are summarized in Table 1. The following components have been investigated for their ability to trigger pyroptosis in glioma cells.

### Temozolomide

Temozolomide (TMZ) is a Food and Drug Administration (FDA)-approved chemotherapeutic agent for the treatment of recurrent anaplastic astrocytoma (approved in 1999) and newly diagnosed GBM (approved in 2005), and it is commonly used in combination with radiotherapy. TMZ is effective in glioma treatment due to its oral bioavailability and its ability to penetrate the blood-brain barrier. It exerts cytotoxic effects primarily through DNA methylation.^119^^,^^120^ Despite its widespread clinical use, the therapeutic efficacy of TMZ in glioma remains limited. Recent studies have shown that TMZ can also induce pyroptosis in glioma cells, suggesting a potential strategy to overcome the drug resistance frequently observed in glioma therapy.

A study by Liu et al. (2021) demonstrated that TMZ treatment induces pyroptosis in GBM cells in a time-dependent manner through upregulation of GSDMD. Morphologically, TMZ-treated GBM cells exhibited prominent membrane ballooning, a hallmark of pyroptosis. In addition, TMZ increased the expression levels of GSDMD, IL-1β, and cleaved caspase-1. Knockdown of GSDMD reduced IL-1β expression and attenuated TMZ-induced pyroptosis *in vitro*, indicating that TMZ induces pyroptosis via GSDMD upregulation in GBM cell.^111^ In another study (2022), a novel strategy using TMZ-loaded magnetic temperature-sensitive liposomes (TMZ/Fe-TSL) was developed to evaluate its therapeutic effects in GBM. Exposure to an alternating magnetic field (AMF) significantly enhanced GBM cell death and increased ROS production. This treatment upregulated pyroptosis-related markers, including NLRP3, caspase-1, and N-terminal GSDMD, while downregulating caspase-3. These findings suggest that TMZ can promote pyroptosis through ROS generation and activation of inflammatory signaling pathways. Notably, this approach highlights a mechanism distinct from conventional apoptosis-based therapies.^112^ Therefore, triggering pyroptosis through chemotherapy agents offers a promising approach to boost anticancer effects, which may result in better outcomes for patients with treatment-resistant tumors.

### Azole compounds

Azole compounds, particularly benzimidazoles such as flubendazole, mebendazole, and fenbendazole, have gained attention for their ability to induce pyroptosis in GBM cells via the NF-κB/NLRP3/GSDMD pathway. This process involves activation of the NLRP3 inflammasome, leading to caspase-1 activation, GSDMD cleavage, and subsequent induction of pyroptosis. Although azole compounds are primarily recognized for their antifungal activity, their capacity to promote cell death mechanisms such as pyroptosis is of particular interest in GBM treatment. By inducing pyroptosis, these compounds suppress tumor cell proliferation and migration and enhance inflammatory responses within the tumor microenvironment, underscoring their potential as therapeutic agents in GBM.^64^^,^^121^

A 2022 study employed weighted gene co-expression network analysis (WGCNA) of The Cancer Genome Atlas (TCGA) GBM dataset, combined with drug repurposing using the Connectivity Map (CMap), to identify azole compounds with potential anti-GBM activity. Among these, three benzimidazole derivatives—flubendazole, mebendazole, and fenbendazole—demonstrated significant, dose-dependent inhibition of GBM cell proliferation. These compounds suppressed DNA synthesis, migration, and invasion, while modulating key epithelial-mesenchymal transition markers. In addition, they induced G2/M cell-cycle arrest via the p53/p21/cyclin B1 pathway.

Importantly, these benzimidazoles induced pyroptosis in GBM cells through activation of the NF-κB/NLRP3/GSDMD pathway and may also trigger mitochondria-dependent apoptosis. *In vivo*, flubendazole significantly reduced tumor growth in a dose-dependent manner without notable toxicity in a nude mouse xenograft model. Collectively, these findings suggest that benzimidazole compounds represent promising candidates for GBM therapy.^64^ Azole compounds may therefore offer a novel therapeutic strategy by promoting pyroptosis and potentially overcoming resistance to conventional treatments.

### Kaempferol

Kaempferol, a flavonoid found in various edible plants, has demonstrated significant anti-glioma activity, particularly in GBM cells. Previous studies have shown that kaempferol suppresses glioma cell proliferation *in vitro* and inhibits tumor growth *in vivo*.^113^^,^^122^ A recent study further reported that kaempferol induces pyroptosis through activation of key molecular components, including NLRP3, caspase-1, ASC, GSDMD, and IL-18, in gastric cancer cells.^123^

Moreover, a study by Chen et al. (2020) demonstrated that kaempferol induces pyroptosis in glioma cells by increasing ROS levels and reducing mitochondrial membrane potential. Elevated ROS levels activate autophagy, which subsequently triggers pyroptosis, as evidenced by GSDME cleavage and the release of pro-inflammatory cytokines such as IL-1β. Kaempferol treatment significantly increased GSDME cleavage, supporting its role in pyroptosis induction. Furthermore, inhibition of autophagy using 3-methyladenine (3-MA) reduced GSDME cleavage, confirming that kaempferol induces pyroptosis through autophagy regulation in glioma cells. These findings suggest that kaempferol is a promising therapeutic agent for GBM, promoting pyroptosis via ROS-mediated autophagy and potentially enhancing treatment efficacy.^113^

### Melatonin

Melatonin, a hormone primarily known for regulating sleep, has shown significant potential in modulating pyroptosis in glioma cells. Recent studies indicate that melatonin receptors are abundantly expressed in glioma tissues and cell lines. When combined with TMZ, melatonin significantly reduced cell viability compared to TMZ alone, while increasing the pyroptosis rate, ROS levels, and the expression of pyroptosis-related proteins.

Melatonin also decreased Nrf2 expression in a dose- and time-dependent manner. In contrast, TMZ alone activated the Nrf2-ARE pathway, which melatonin was able to inhibit via suppression of the IGF-1/AKT/mTOR signaling pathway. Notably, overexpression of Nrf2 in glioma cells attenuated the increase in pyroptosis-related proteins induced by melatonin, indicating that melatonin-mediated pyroptosis depends, at least in part, on Nrf2 inhibition. *In vivo* studies further demonstrated that the combination of melatonin and TMZ significantly reduced tumor size and increased pyroptosis without adversely affecting body weight or liver function in mice. Given the limited efficacy of TMZ monotherapy, melatonin may serve as a promising chemosensitizer, enhancing therapeutic outcomes in GBM by promoting pyroptosis and modulating key signaling pathways.^114^

### Dimethoxycanthin-6-one

Dimethoxycanthin-6-one, particularly the 4,5-dimethoxy variant, has been identified as a novel lysine-specific demethylase 1 (LSD1) inhibitor and shows significant potential in treating GBM. Research indicates that this compound not only inhibits the proliferation of GBM cells but also induces both apoptosis and pyroptosis. In studies involving GBM cell lines such as U251 and T98G, treatment with 4,5-dimethoxycanthin-6-one resulted in increased expression of key markers associated with pyroptosis, including NLRP3, caspase-1, IL-1β, and IL-18. These findings suggest that the compound activates the pyroptosis signaling pathway, enhancing inflammatory responses and potentially contributing to tumor suppression. Moreover, *in vivo* experiments demonstrated that 4,5-dimethoxycanthin-6-one significantly reduced tumor growth in animal models, indicating its therapeutic potential.^115^ In conclusion, the role of dimethoxycanthin-6-one in promoting pyroptosis presents a novel approach to glioma management, warranting further investigation into its mechanisms and clinical applications.

### AT7519

AT7519, a cyclin-dependent kinase inhibitor, has demonstrated significant potential in treating GBM by inducing apoptosis and pyroptosis in cancer cells. Research indicates that AT7519 exerts its antitumor effects through several mechanisms. It arrests the cell cycle at the G1 and G2 phases, inhibits GBM cell proliferation, and activates pyroptosis via the caspase-3-mediated cleavage of GSDME. In studies involving GBM cells, treatment with AT7519 resulted in observable morphological changes consistent with pyroptosis, including membrane ballooning protrusions and increased lactate dehydrogenase (LDH) release, indicating compromised membrane integrity. The activation of pyroptosis was confirmed by the elevation of the GSDME-N in a dose-dependent manner, highlighting the role of caspase-3 in this process. The induction of pyroptosis by AT7519 contributes to the direct cytotoxic effects on GBM cells and enhances the inflammatory response, potentially improving the overall therapeutic efficacy against this aggressive cancer. Due to this dual mechanism of inducing apoptosis and pyroptosis, AT7519 is a promising candidate for GBM treatment, particularly in overcoming the challenges associated with conventional therapies and tumor resistance.^96^ In conclusion, the potential of AT7519 to stimulate pyroptosis in GBM cells represents a novel approach in cancer therapy. This underscores the need for further investigation into its clinical applications and mechanisms of action, which could lead to more effective GBM treatments.

### EZH2 inhibitor

Enhancer of zeste homolog 2 (EZH2) is a critical component of the polycomb repressive complex 2 (PRC2). This complex mediates histone methylation, particularly the trimethylation of lysine 27 on histone H3 (H3K27me3), leading to gene silencing. This epigenetic modification regulates genes involved in cell proliferation, migration, and invasion genes.^124^^,^^125^ EZH2 serves a crucial function as an oncogenic factor in gliomas, primarily by suppressing pyroptosis. Studies have investigated its involvement in apoptosis, autophagy, and the regulation of the cell cycle in GBM.^126^^,^^127^

EZH2 inhibitors, particularly 3-deazaneplanocin A (DZNep), have shown promising potential in inducing pyroptosis in GBM. A recent study from 2024 shows the significant role of EZH2 in regulating pyroptosis through the EZH2-STAT3 signaling pathway. *In vitro*, experiments demonstrated that DZNep effectively triggers pyroptosis in GBM cells by increasing the levels of crucial pyroptotic factors, specifically NLRP3 and GSDMD. Furthermore, treatment with DZNep, in combination with the STAT3 inhibitor, resulted in further increase in these pyroptosis-related factors. The simultaneous inhibition of both EZH2 and STAT3 promoted pyroptosis and led to the increased expression of inflammatory cytokines such as IL-1β and IL-18. These findings suggest that EZH2 regulates pyroptosis in GBM via the STAT3 pathway, indicating that targeting this mechanism could be a viable strategy for immunotherapy in treating GBM.^74^

### Xihuang pill

Xihuang pill (XHP), also known as Xihuang Wan, is a traditional Chinese medicine that has been used for centuries in China for the treatment of various cancers.^128^^,^^129^ XHP has been identified as a potential agent for inducing pyroptosis, particularly in breast cancer cells.^130^ Research shows that the XHP induces pyroptosis in glioma cells by modulating the POU4F1/STAT3 axis. Treatment with XHP downregulates both POU4F1 and STAT3, resulting in pyroptotic changes such as cell swelling and ballooning, increased LDH release, and elevated pro-inflammatory cytokines like IL-1β and IL-18. POU4F1 serves as a transcription factor for STAT3. So, its overexpression or STAT3 silencing can inhibit the pyroptotic effects of XHP. Recent studies have shown that XHP induces pyroptosis in glioma cells by modulating the POU4F1/STAT3 axis. Treatment with XHP downregulates both POU4F1 and STAT3, resulting in pyroptotic morphological changes such as cell swelling and ballooning, increased lactate LDH release, and elevated levels of pro-inflammatory cytokines, including IL-1β and IL-18. POU4F1 functions as a transcriptional regulator of STAT3; therefore, overexpression of POU4F1 or silencing of STAT3 can attenuate the pyroptotic effects induced by XHP.

Moreover, XHP activates the NLRP3 inflammasome in glioma cells. *In vitro* studies have demonstrated that XHP reduces cell viability while increasing the number of pyroptotic cells and upregulating pyroptosis-related proteins such as cleaved caspase-1 and IL-1β. *In vivo* experiments further confirm that XHP promotes pyroptosis and suppresses tumor growth in glioma models.^116^

### Arsenic compounds

Several studies have demonstrated that arsenic compounds, particularly arsenic trioxide (ATO), significantly induce pyroptosis.^131^^,^^132^^,^^133^ A recent study demonstrated that ATO induces pyroptosis in glioma cells. ATO treatment leads to significant cleavage of GSDME, with caspase-3 activation essential for this process. Upon exposure to ATO, glioma cells show characteristic signs of pyroptosis, including increased LDH release and morphological changes like cell swelling and membrane rupture, confirmed by transmission electron microscopy. Additionally, ATO may activate inflammasomes, contributing to the inflammatory response associated with pyroptosis. These findings suggest that arsenic compounds could serve as potential therapeutic agents in glioma treatment by leveraging their ability to induce pyroptosis and promote tumor cell death.^107^

### Galangin

Galangin, a natural flavonoid found in various plants, has garnered attention for its potential antitumor properties, particularly in recent studies demonstrating its ability to induce pyroptosis. A study by Yang Kong and colleagues in 2019 showed that galangin could inhibit the viability and proliferation of human GBM cell lines in a dose-dependent manner by inducing pyroptosis in these cells. This process involves the cleavage of GSDME. Morphological assessments with phase-contrast microscopy revealed large bubbles on the cell membrane of dying cells and swelling typical of pyroptosis. Additionally, there was a significant increase in LDH release in GBM cells, indicating that galangin treatment compromised the integrity of the plasma membrane in these tumor cells.^108^

### Isobavachalcone

Isobavachalcone (IBC), a flavonoid derived from *Psoralea corylifolia*, has attracted attention for its potential therapeutic effects in glioma, particularly its role in inducing pyroptosis. A 2022 study investigated the effects of IBC in GBM and elucidated its antitumor mechanisms. The researchers found that IBC effectively suppressed the proliferation, migration, and invasion of GBM cells *in vitro* and inhibited tumor growth *in vivo* without significant toxicity in GBM xenograft models. IBC targets the NLRP3 inflammasome pathway through modulation of estrogen receptor α (ESR1). In addition, IBC reduced NLRP3-inflammasome-related pyroptosis and inflammation and induced cell-cycle arrest at the G1 phase. Furthermore, the inhibitory effects of IBC on NLRP3 could be reversed by the NLRP3 antagonist CY-09 in both *in vivo* and *in vitro* experiments. These findings suggest that IBC is a potential therapeutic agent against GBM and provides new insights into treatment strategies for this aggressive cancer.^117^

### Aloe-emodin

Aloe-emodin (AE) is an anthraquinone compound shown to influence pyroptosis through various mechanisms. Studies have indicated that AE induces pyroptosis in HeLa cells, a cerebral ischemia-reperfusion injury model, and diabetic cardiomyopathy.^134^^,^^135^^,^^136^ Recently, a study demonstrated that AE significantly induces pyroptosis in GBM cells. This compound triggers cell death in GBM tumor cells in a time- and concentration-dependent way, primarily through activating the caspase-3/GSDME pyroptosis pathway. Additionally, the morphology of the AE-treated GBM cells exhibited characteristics specific to pyroptosis, such as swelling of the cell membrane and blebbing, indicating that Aloe-emodin may trigger pyroptosis in these cells. Moreover, measurements of LDH release from AE-treated U87MG cells revealed an increase in LDH levels as the concentration of AE dose. These findings suggest that AE inhibits cell proliferation while inducing pyroptosis in GBM cells.^109^

### Lidocaine

Lidocaine, a widely used local anesthetic, plays a significant role in inducing pyroptosis in GBM cells. A study demonstrated that lidocaine exposure leads to an increase in cytosolic calcium levels in GBM cells. This calcium influx is mediated through activation of Ca^2+^/calmodulin-dependent protein kinase II (CaMKII). At concentrations such as 2 mM, lidocaine enhances cytosolic calcium flux and upregulates the expression of caspase-3 and GSDME. Importantly, silencing the CaMKII gene using siRNA significantly inhibits these effects, indicating that CaMKII activation is essential for lidocaine-induced pyroptosis. Mechanistically, lidocaine activates CaMKII, which phosphorylates TRPV1 ion channels, resulting in calcium overload in GBM cells. This overload subsequently triggers the pyroptotic pathway. Lidocaine-induced pyroptosis contributes to its antiproliferative effects on GBM cells by inhibiting tumor growth and promoting inflammatory responses that may help suppress tumor progression.^110^

### Simvastatin

Simvastatin, a widely used cholesterol-lowering statin, has been found to regulate pyroptosis, particularly in cancer cells such as colon, non-small cell lung, and gastric cancer. Its role in pyroptosis induction involves activation of the ROS/NLRP3/caspase-1/GSDMD axis as well as the caspase-3/GSDME pathway.^137^^,^^138^^,^^139^ On the other hand, a recent study showed that simvastatin inhibited GBM progression by inducing caspase-1-dependent pyroptosis. The results demonstrated that simvastatin treatment significantly reduced the viability of GBM cells and suppressed their migratory and invasive capabilities in a dose-dependent manner. Notably, simvastatin suppressed key markers of pyroptosis, including caspase-1, NLRP3, and IL-1β, suggesting that the drug inhibits caspase-1-dependent pyroptosis in GBM cells. This finding is notable because pyroptosis can have dual roles in cancer, sometimes promoting inflammation that supports tumor growth and other times acting as a tumor-suppressive mechanism. Thus, simvastatin’s inhibition of pyroptosis appears to contribute to its anti-tumor effects by reducing inflammatory signaling that may facilitate tumor progression in GBM cells.^118^

The contrasting effects of simvastatin on pyroptosis regulation in different cancer cell types highlight the complexity and context dependency of pyroptosis in cancer biology. Simvastatin can induce pyroptosis and cell death in certain cancers, whereas in GBM, its antitumor effects are associated with inhibition of pyroptosis and related inflammatory pathways. These findings emphasize the importance of tumor-specific investigations when considering the therapeutic use of agents such as simvastatin and the targeting of pyroptosis in cancer treatment.

### Regulatory roles of noncoding RNAs in pyroptosis of glioblastoma multiforme

Noncoding RNAs (ncRNAs) are functional RNA molecules transcribed from DNA that are not translated into proteins and are involved in the regulation of gene expression. ncRNAs have emerged as key regulators of pyroptosis, particularly in GBM, by modulating the expression and activity of pyroptosis-related genes and pathways. Several ncRNAs, including long noncoding RNAs (lncRNAs) and microRNAs (miRNAs), influence pyroptosis by targeting components such as inflammasomes, caspases, and GSDMs.^140^^,^^141^

Accumulating evidence indicates that ncRNAs are not only key regulators of gene expression in GBM but also promising diagnostic biomarkers and therapeutic targets.^142^^,^^143^ Aberrant expression of miRNAs and lncRNAs has been consistently associated with GBM initiation, progression, treatment resistance, and patient prognosis.^144^^,^^145^ Importantly, these ncRNAs primarily exert their functions through complex mRNA–ncRNA regulatory networks, including competing endogenous RNA (ceRNA) interactions, which finely regulate the expression of oncogenes, tumor suppressors, and cell death–related genes, including those involved in pyroptosis.^146^

Recent studies have further highlighted pyroptosis-related mRNA-miRNA-long non-coding RNA (lncRNA) networks that are closely associated with GBM prognosis, immune infiltration, and therapeutic response.^144^^,^^147^^,^^148^^,^^149^^,^^150^ For example, integrative analyses using TCGA and GTEx datasets identified key pyroptosis-related genes (CASP3, CASP9, GZMB, NLRP2, and TP63) and constructed prognostic models that effectively stratified GBM patients into distinct risk groups with significantly different survival outcomes. These models were further linked to immune microenvironment characteristics and inflammatory pathway activity, underscoring the functional relevance of pyroptosis-associated regulatory networks.^147^

In addition, ceRNA network analyses have identified specific regulatory axis, such as CASP3/TP63–miR-519c-5p–GABPB1-AS1, suggesting that ncRNAs can modulate core pyroptosis executors at the mRNA level. Experimental validation of pyroptosis-related lncRNAs, such as COX10-AS1, further confirmed their role in regulating CASP1, NLRP3, IL-1β, and IL-18 expression and influencing GBM cell proliferation and inflammatory cell death.^151^ Collectively, these findings demonstrate that mRNA-ncRNA interaction networks play a central role in regulating pyroptosis and shaping GBM progression, immune responses, and therapeutic sensitivity.

### MicroRNAs

miRNAs play critical roles in post-transcriptional gene regulation, including modulating cell death pathways such as pyroptosis. Recent evidence indicates that miRNAs can influence this pathway in GBM tumors.^152^ One well-characterized example is miR-214. A study by Jiang et al. revealed that miR-214 modulates the expression of key genes in the pyroptotic pathway, thereby impacting tumor progression. In GBM tissues and cell lines, miR-214 is significantly downregulated, whereas caspase-1 is upregulated, indicating that miR-214 negatively regulates caspase-1 and influences pyroptosis. The study found that miR-214 suppresses GBM cell proliferation and migration by targeting caspase-1. Additionally, cells transfected with a miR-214 mimic showed a significant reduction in mRNA levels of IL-18 and IL-1β; however, this decrease was reversed by subsequent transfection with a miR-214 inhibitor. Low levels of miR-214 lead to increased caspase-1 activity, promoting pyroptosis and potentially enhancing inflammatory responses that affect tumor behavior. These findings demonstrate the critical role of miR-214 in regulating pyroptosis and its implications for GBM progression, highlighting its potential relevance for future research in this area.^153^

Moreover, another study revealed that simvastatin inhibits GBM progression by suppressing caspase-1-dependent pyroptosis regulated by miR-214. The results showed that miR-214 upregulation significantly reduces the expression of key pyroptosis markers, including caspase-1, NLRP3, and IL-1β. Thus, miR-214 negatively regulates these markers and inhibits the pyroptosis pathway in GBM cells. The study also indicated that the antitumor effects of simvastatin, including reduced cell proliferation, migration, and invasion, are closely associated with this miR-214-mediated suppression of pyroptosis. When miR-214 is inhibited, the expression of caspase-1, NLRP3, IL-1β, and IL-18 increases, reversing the effects of simvastatin and enhancing pyroptotic signaling. Conversely, miR-214 overexpression suppresses these markers and strengthens the antitumor effects of simvastatin.^118^

In conclusion, miRNAs act as essential regulators of pyroptosis in GBM by targeting key pyroptotic components, such as caspase-1 and inflammasome proteins. These findings demonstrate the therapeutic potential of specific miRNAs in modulating pyroptosis and influencing GBM progression.

### Long non-coding RNAs

LncRNAs add an additional layer of regulatory complexity by functioning as ceRNAs, scaffolds, transcriptional regulators, or epigenetic modulators.^154^ Through miRNA sponging, lncRNAs can indirectly regulate the expression of pyroptosis-related mRNAs. For instance, oncogenic lncRNAs may sequester tumor-suppressive miRNAs that normally inhibit inflammasome components or caspases, thereby suppressing pyroptosis and promoting tumor progression. Conversely, tumor-suppressive lncRNAs can enhance pyroptosis by relieving miRNA-mediated repression of key mRNAs involved in inflammatory cell death pathways.^140^

Emerging studies have indicated the critical regulatory roles of lncRNAs in modulating pyroptosis to influence GBM progression and patient outcomes. Some pyroptosis-related lncRNAs (PRlncRNAs) act as oncogenic drivers by promoting inflammatory pathways. Conversely, protective lncRNAs suppress tumorigenesis by dampening pyroptosis-associated immune activation. These lncRNAs co-express with key pyroptosis-related genes, forming prognostic signatures.^155^^,^^156^

A recent study identified a pyroptosis-related lncRNA signature (PRLS) in GBM, demonstrating that lncRNAs critically regulate pyroptosis by modulating inflammatory pathways and immune evasion. By analyzing large patient cohorts from the TCGA and CGGA databases, the researchers found that high-risk patients, characterized by upregulated oncogenic lncRNAs and downregulated tumor-suppressive lncRNAs, exhibited shorter survival, chemotherapy resistance, and an immunosuppressive microenvironment with elevated immune checkpoint expression. The PRLS accurately predicted outcomes across cohorts and highlighted lncRNAs as dual regulators of pyroptosis, promoting either anti-tumor immunity or tumor progression, as well as potential biomarkers for prognosis and therapy. Therefore, lncRNAs play a critical and complex role in regulating pyroptosis in GBM, with their effects varying depending on specific lncRNA interactions and the tumor microenvironment. Understanding these mechanisms may enable the development of novel therapeutic strategies targeting lncRNAs to modulate pyroptosis and improve clinical outcomes.^149^

Moreover, a study by Tanzhu and colleagues in 2022 identified 23 pyroptosis-related lncRNAs using data from The TCGA and the CGGA. The researchers established a prognostic signature comprising 13 lncRNAs through Pearson correlation analysis. They classified glioma patients into high-risk and low-risk groups based on their expression profiles, finding that those in the high-risk group had significantly lower survival probabilities. The study showed that the established signature predicts patient outcomes and reflects the immune landscape and molecular alterations associated with different risk groups, suggesting that pyroptosis-related lncRNAs can serve as valuable biomarkers for glioma prognosis.^157^

Another study focused on constructing a pyroptosis-related lncRNA signature to predict prognosis and responses to immunotherapy in glioma patients. The study utilized LASSO regression to identify key lncRNAs associated with patient outcomes. The researchers identified PRLs and constructed a prognostic model that predicts patient outcomes and reflects the immune microenvironment and mutation landscape. The results showed that some lncRNAs are upregulated in glioma and can inhibit pyroptosis, contributing to tumor progression and poor prognosis. In contrast, others are downregulated and may promote pyroptosis, suppressing tumor growth. The constructed lncRNA signature divides patients into high- and low-risk groups, with high-risk patients showing lower survival and distinct immune infiltration patterns compared with low-risk patients. The findings indicate that specific lncRNAs can modulate pyroptosis pathways in glioma cells.^150^

A study in 2023 also performed an integrative analysis using data from TCGA and CGGA to identify novel pyroptosis-related lncRNAs linked to clinical outcomes in glioma patients. The researchers established distinct molecular subtypes of gliomas by employing consensus clustering based on pyroptosis-related genes. They found significant differences in immune infiltration, somatic copy-number alterations, and mutations between high-risk and low-risk groups. Identifying these lncRNAs contributes to understanding the regulatory networks involved in glioma progression.^158^ Moreover, Wu and colleagues utilized machine learning techniques to develop a model based on pyroptosis-related lncRNAs, aiming to improve prognostic prediction in low-grade glioma patients. The model integrated multiple data types to enhance the accuracy of outcome prediction. It identified key lncRNAs associated with pyroptotic processes and provided insights into potential therapeutic targets and strategies for managing low-grade gliomas.^159^

Together, these findings show the dual and context-dependent roles of lncRNAs in regulating pyroptosis and shaping the immune microenvironment in GBM. From a translational perspective, ncRNAs represent promising diagnostic and prognostic biomarkers due to their stability in tissues, serum, cerebrospinal fluid, and extracellular vesicles, making them suitable for non-invasive disease monitoring.^160^ Importantly, the specificity of mRNA-ncRNA interactions enables precise regulation of disease-relevant pathways.

Emerging evidence indicates that ncRNAs function within complex regulatory networks, including ceRNA systems, in which lncRNAs, miRNAs, and mRNAs interact to modulate gene expression. These mRNA-ncRNA interaction networks play a critical role in controlling pyroptosis-related pathways and tumor progression in GBM. Therapeutic strategies targeting these networks, such as miRNA mimics or lncRNA silencing approaches, hold significant promise for restoring pyroptotic signaling while minimizing off-target effects.^140^

However, the mRNA-ncRNA regulatory landscape in GBM-associated pyroptosis remains incompletely defined. Future studies should prioritize the systematic construction and experimental validation of these interaction networks to identify key regulatory nodes and functional axes. A deeper understanding of these integrated networks will provide important mechanistic insights into pyroptosis regulation and support the development of more precise and effective therapeutic strategies for GBM.

Although numerous pharmacological agents and ncRNAs have been reported to induce pyroptosis in GBM, their broader implications for therapeutic development require deeper consideration. Current evidence indicates that these agents largely converge on a limited set of core molecular axis, including inflammasome activation (NLRP3-caspase-1-GSDMD), oxidative stress signaling, and the caspase-3-GSDME pathway. This convergence suggests that future strategies may focus on optimizing or repurposing compounds targeting these shared execution pathways rather than relying solely on novel drug discovery. Among these, GSDMDs, particularly GSDMD and GSDME, emerge as central therapeutic targets. GSDME expression appears to determine whether tumor cells undergo apoptosis or pyroptosis in response to treatment. Therefore, restoring or upregulating GSDME through epigenetic modulation, ncRNA-based regulation, or combination therapies may redirect apoptosis-resistant cells toward pyroptosis and overcome therapeutic resistance.

ncRNAs further refine this landscape by acting as key regulators of pyroptosis-related networks, modulating inflammasomes, caspases, GSDMs, and oncogenic pathways such as NF-κB, STAT3, and PI3K/AKT. These findings show their potential not only as biomarkers but also as therapeutic targets using approaches such as miRNA mimics, inhibitors, or lncRNA silencing. Combination strategies represent another promising direction. Pyroptosis-inducing agents may act as sensitizers to enhance the efficacy of standard treatments, including TMZ, radiotherapy, and immunotherapy, by lowering the threshold for inflammatory cell death and overcoming resistance mechanisms.

Future research should prioritize identifying upstream regulators of GSDM activation, mapping pyroptosis-related mRNA-ncRNA interaction networks, and developing delivery systems capable of crossing the blood-brain barrier. Functional validation in clinically relevant models, including patient-derived organoids and immunocompetent systems, will be essential to enable safe and effective clinical translation.

### Nucleic-acid-based therapeutic strategies and delivery challenges in glioblastoma multiforme

Nucleic-acid-based therapeutics represent a promising strategy to reprogram GBM cells by directly modulating oncogenic signaling, treatment resistance, and tumor-associated regulatory networks at genetic and epigenetic levels.^161^^,^^162^ These approaches include miRNA mimics and inhibitors, small interfering RNAs (siRNAs), antisense oligonucleotides, and lncRNA-targeting constructs, enabling precise regulation of disease-driving genes, including those involved in pyroptosis.^140^^,^^149^^,^^163^ They can selectively target oncogenic transcripts, restore tumor-suppressive ncRNAs, and effectively modulate pyroptosis-related pathways that are often difficult to target with conventional pharmacological agents.^140^^,^^141^^,^^143^^,^^164^ Despite their therapeutic potential, clinical translation is limited by delivery barriers, particularly the blood-brain barrier. To overcome this, several delivery platforms have been developed. Nanoparticle-based systems, including lipid nanoparticles and polymeric carriers, protect nucleic acids from degradation and enhance cellular uptake. Surface modification with ligands such as transferrin or angiopep-2 enables receptor-mediated blood-brain barrier transcytosis and tumor targeting.^165^^,^^166^^,^^167^

Exosome-based delivery systems have emerged as promising platforms for nucleic acid therapeutics in GBM, particularly for overcoming blood-brain barrier limitations. Their biocompatibility and low immunogenicity allow them to evade immune clearance and utilize endogenous transport mechanisms for brain delivery. Engineered exosomes can carry diverse nucleic acid cargos targeting pyroptosis-related regulators. Beyond delivery, they may modulate the tumor microenvironment and enhance inflammatory responses, making them attractive tools for precise and sustained activation of pyroptotic pathways in GBM.^168^^,^^169^^,^^170^^,^^171^^,^^172^^,^^173^

Viral vectors, including adeno-associated viruses and lentiviruses, are effective tools for stable delivery of genes and ncRNAs in GBM, enabling transduction of both dividing and non-dividing cells. However, their clinical application is limited by immunogenicity, safety concerns, and restricted cargo capacity.^174^^,^^175^^,^^176^ To overcome blood-brain barrier constraints, complementary strategies have been developed. Convection-enhanced delivery allows direct intratumoral administration, achieving high local concentrations.^177^^,^^178^ In addition, stimuli-responsive nanoparticles and focused ultrasound with microbubbles enhance blood-brain barrier permeability and enable targeted, controlled release of nucleic acid therapeutics within GBM tissue.^179^^,^^180^^,^^181^^,^^182^^,^^183^

Collectively, these strategies provide complementary approaches to overcome blood-brain barrier limitations and improve nucleic acid delivery to GBM. Integration of these delivery platforms with ncRNA-based regulation of pyroptosis represents a promising direction for precision GBM therapy.

### Dual role and clinical implications of pyroptosis in GBM

Although pyroptosis has emerged as a promising strategy to overcome apoptosis resistance in GBM, its biological effects are highly context-dependent and require careful consideration. Pyroptosis is an inflammatory form of programmed cell death characterized by GSDM-mediated membrane pore formation and the release of pro-inflammatory cytokines such as IL-1β and IL-18. In apoptosis-resistant GBM, pyroptosis can eliminate tumor cells that evade caspase-dependent apoptotic pathways. It also promotes the release of DAMPs, enhances tumor immunogenicity, and facilitates recruitment of cytotoxic immune cells, including CD8^+^ T cells and natural killer cells, thereby potentially shifting the immunosuppressive GBM microenvironment toward an immune-active state. However, pyroptosis-associated inflammation may also have harmful consequences. In the confined intracranial space, excessive or sustained inflammatory responses can lead to neuroinflammation, cerebral edema, and injury to surrounding healthy brain tissue. Chronic inflammation may also paradoxically promote tumor progression by enhancing angiogenesis, supporting glioma stem cell maintenance, and activating pro-survival pathways such as NF-κB and STAT3. Additionally, inflammatory cytokines may recruit immunosuppressive myeloid cells, thereby weakening anti-tumor immunity. These opposing effects highlight the dual nature of pyroptosis in GBM. Tumor heterogeneity, including variability in gasdermin expression and inflammasome activation, further influences therapeutic outcomes. Moreover, cross-talk among pyroptosis, apoptosis, and necroptosis pathways may determine whether inflammatory cell death suppresses or promotes tumor progression. Therefore, precise spatial and temporal regulation is essential to harness pyroptosis safely and effectively while minimizing neuroinflammatory toxicity.^184^^,^^185^^,^^186^

Beyond inflammatory balance, reliable biomarkers are needed to predict and monitor pyroptosis activation in GBM. Expression profiles of GSDMs, inflammasome components, caspases, and cytokines may serve as potential diagnostic and prognostic indicators. Integration with ncRNA-based signatures may further improve patient stratification. MicroRNAs and lncRNAs regulate inflammasomes, caspases, GSDMs, and key signaling pathways such as NF-κB, STAT3, and AKT/mTOR, offering opportunities for precise modulation of pyroptosis through ncRNA-mRNA networks. From a therapeutic perspective, combining pyroptosis-inducing strategies with standard treatments such as TMZ, radiotherapy, and immunotherapy represents a promising approach. Coordinated activation of apoptosis and pyroptosis, particularly when combined with nucleic-acid-based therapies delivered via blood-brain barrier-penetrating systems, may enhance tumor eradication and reduce therapeutic resistance.

## Conclusion

The study of GBM treatment revealed an essential understanding of the difficulties in overcoming this aggressive brain cancer. Despite the standard therapies, the outcomes remain unsatisfactory, largely because of the tumor’s resistance strategies and its highly invasive characteristics. Pyroptosis is a promising alternative pathway of cell death that could overcome the resistance of GBM cells. Consequently, regulating signaling pathways that modulate pyroptosis could overcome this resistance or enhance treatment approaches. Persistent investigation into the mechanisms of pyroptosis and its function in GBM therapy might improve patient outcomes and prolong survival rates.

### Future perspectives and challenges

Despite growing evidence supporting pyroptosis as a promising therapeutic mechanism in GBM, several critical challenges must be addressed before its clinical translation can be achieved. Although pyroptosis offers a potential strategy to overcome apoptosis resistance, its biological effects are highly context-dependent. In particular, pyroptosis-driven inflammation may exert detrimental effects within the confined intracranial environment. Therefore, precise spatial and temporal regulation of pyroptosis is essential to maximize therapeutic benefit while minimizing harmful neuroinflammation.

Beyond these biological challenges, limited delivery efficiency across the blood-brain barrier and the risk of systemic toxicity remain significant challenges. Future research should focus on identifying reliable biomarkers, optimizing combination strategies, and developing targeted delivery systems, particularly for nucleic-acid-based therapeutics, to improve tumor specificity. Translation into clinical practice will also require well-designed preclinical models, patient stratification strategies, and early-phase clinical trials.

Moreover, most current evidence is derived from *in vitro* studies, bioinformatics analyses, or immunodeficient animal models, which do not fully recapitulate the human tumor microenvironment. Species-specific differences in pyroptotic responses further complicate interpretation. Therefore, future work should prioritize immunocompetent models, patient-derived organoids, and advanced three-dimensional systems. Ensuring safety and controllability will be critical, as excessive pyroptosis may cause neuroinflammation and systemic toxicity. Developing strategies that enable precise regulation of pyroptosis will be essential for safe and effective clinical application.

Finally, an additional emerging area is the potential interaction between RNA-based immunotherapies, such as RNA dendritic cell vaccines, and pyroptotic pathways. To date, there is no direct experimental evidence showing that these vaccines induce or regulate pyroptosis in GBM. However, because RNA sensing pathways can activate innate immune and inflammasome signaling, an indirect relationship cannot be excluded. Future studies should investigate whether RNA-based vaccines influence GSDM activation, inflammasome assembly, or pyroptosis-associated cytokine release within the GBM microenvironment.

## Acknowledgments

We thank the Neuroscience Institute, Imam Khomeini Hospital Complex, Tehran University of Medical Sciences, Tehran, Iran. The authors have not received financial support for the research, authorship, and publication of this study.

## Author contributions

S.K., A.R.D., M.H., A.K., R.A.Y., and M.A. were involved in data collection and drafting the manuscript. H.M. and S.H. played a role in the conception and design of the manuscript and provided critical revisions. All authors gave their approval for the final version of the manuscript.

## Declaration of interests

The authors declare no conflict of interests.
